# Case Report: Not all signet rings are of gastric origin: a case of lobular breast carcinoma metastatic to the stomach

**DOI:** 10.3389/fonc.2026.1852381

**Published:** 2026-06-12

**Authors:** Amalia A. Sofianidi, Constantinos Papadimitris, Maria Papanikolaou, Eleftheria Lakiotaki, Penelope Korkolopoulou, Meletios-Athanasios Dimopoulos, Maria Kaparelou

**Affiliations:** 1 Eugenideio Hospital, National and Kapodistrian University of Athens, Athens, Greece; 2 First Department of Pathology, Laikon General Hospital, Medical School, National and Kapodistrian University of Athens, Athens, Greece; 3 Department of Clinical Therapeutics, “Alexandra” General Hospital, Medical School of National and Kapodistrian University of Athens, Athens, Greece

**Keywords:** breast cancer, gastric metastasis, GATA3, immunohistochemistry, invasive lobular carcinoma, signet ring, TRPS1

## Abstract

**Background:**

Invasive lobular carcinoma (ILC) is a distinct breast cancer subtype with a characteristic infiltrative growth pattern linked to loss of E-cadherin. GI metastases may clinically and histologically mimic primary GI malignancies, creating significant diagnostic pitfalls.

**Case presentation:**

A 52-year-old woman with *de novo* metastatic, grade 1, luminal A ILC (ER+/PR+, HER2 0; Ki-67 5%) diagnosed in 2022 achieved durable disease control with systemic therapy and maintenance ribociclib plus ovarian suppression. In late 2025, she developed progressive epigastric discomfort and rising tumour markers. Gastroduodenal endoscopy demonstrated erythematous gastric mucosa with focal whitish areas. Biopsies showed diffuse-type adenocarcinoma with signet ring morphology, initially interpreted as primary gastric cancer. Expert review with comparative pathology and extended immunohistochemistry demonstrated GATA3 and TRPS1 positivity, strong ER/PR expression, CDX2 negativity, and loss of E-cadherin, supporting gastric metastasis from ILC. Despite no clear radiologic progression on CT, symptomatic gastric involvement and weight loss prompted initiation of second-line weekly paclitaxel.

**Conclusion:**

Signet ring morphology in gastric biopsies is not pathognomonic for primary gastric carcinoma. In patients with current or prior ILC, an IHC panel incorporating breast-lineage markers (e.g., GATA3, TRPS1) and GI markers (e.g., CDX2, CK20), alongside E-cadherin status, is pivotal to avoid misdiagnosis, inappropriate surgery, and delay of systemic therapy.

## 1 Introduction

Gastric metastasis from breast cancer is a relatively rare event. One single-institution review reporting an incidence of 0.04% (5/14,169 breast cancer cases) (1). However, invasive lobular carcinoma (ILC) is disproportionately represented: in a landmark series of 51 patients with gastric metastases from breast cancer, lobular histology accounted for 70% (36/51) of cases, far exceeding its ~10–15% share of all breast cancers (2). Indeed, gastrointestinal (GI) metastases are far more common in ILC (~4.5%) than in invasive ductal carcinoma (IDC) (~0.2%) (3). The susceptibility of ILC to metastasize to the GI tract has been described for years; the lack of the E‐cadherin protein expression facilitates diffuse infiltration of serosal and mucosal surfaces (4). Gastric involvement can mimic primary diffuse-type gastric carcinoma, including linitis plastica–like infiltration or, more rarely, signet ring morphology—an appearance classically associated with primary gastric cancer.

Signet ring cell morphology in gastric metastases from ILC is the principal characteristic that creates an almost flawless histopathologic mimic of primary diffuse gastric adenocarcinoma, with deep diagnostic and therapeutic consequences. Signet ring cell differentiation is not always present in the primary breast tumor, rather it may emerge or become predominant only in the metastatic setting. In a classic clinicopathologic study of ten ILC cases with signet ring features, two primary tumors lacked signet ring cells entirely, yet the metastases showed prominent signet ring morphology, attesting to the morpho-functional heterogeneity of lobular carcinoma (5). Notably, the World Health Organization (WHO) classification does not recognize signet ring differentiation in breast carcinoma as a discrete entity, and significant disparities between the primary tumor and its signet ring component, including molecular subtype shifts, have been documented (6).

## 2 Case presentation

A 52-year-old female was diagnosed in 2022 with *de novo* metastatic breast cancer after presenting with malignant ascites. Ovarian cancer was initially suspected; however, cytologic evaluation demonstrated metastatic adenocarcinoma. Immunohistochemistry (IHC) was negative for PAX-8, p53, WT-1, CDX-2, CEA, and calretinin, while positive for GATA-3 and estrogen receptor (ER). Based on these findings, a breast primary was suspected. Subsequent biopsy confirmed a grade 1 ILC, luminal A subtype (ER-positive, progesterone receptor [PR]-positive, human epidermal growth factor receptor 2 [HER2] score 0), with low proliferative activity (Ki-67 = 5%). Baseline imaging revealed bone metastases and multiple peritoneal implantations. No gastric pathology was identified on imaging performed at the time of the initial breast cancer diagnosis. The patient received first-line treatment therapy with carboplatin and paclitaxel, achieving marked clinical and radiologic improvement.

In July 2022, maintenance therapy with ribociclib plus ovarian suppression (GnRH analog) was initiated, along with intravenous zoledronic acid. The patient remained clinically stable with durable disease control.

**Figure 1:**
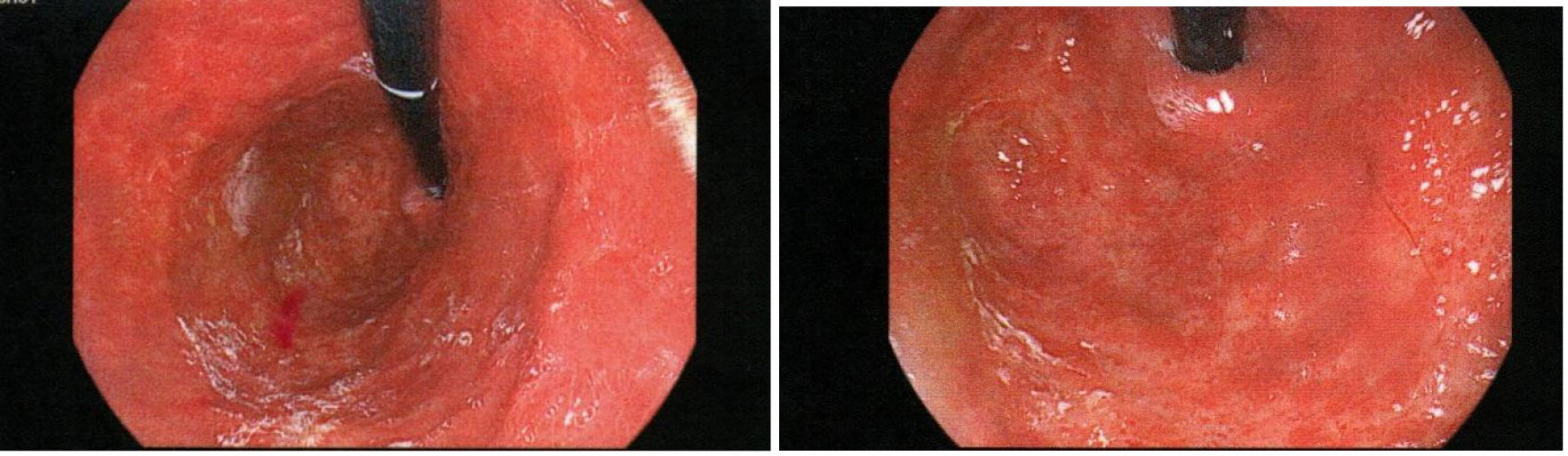
Gastroduodenal endoscopy shows markedly erythematous gastric mucosa with focal whitish areas.

**Figure 2:**
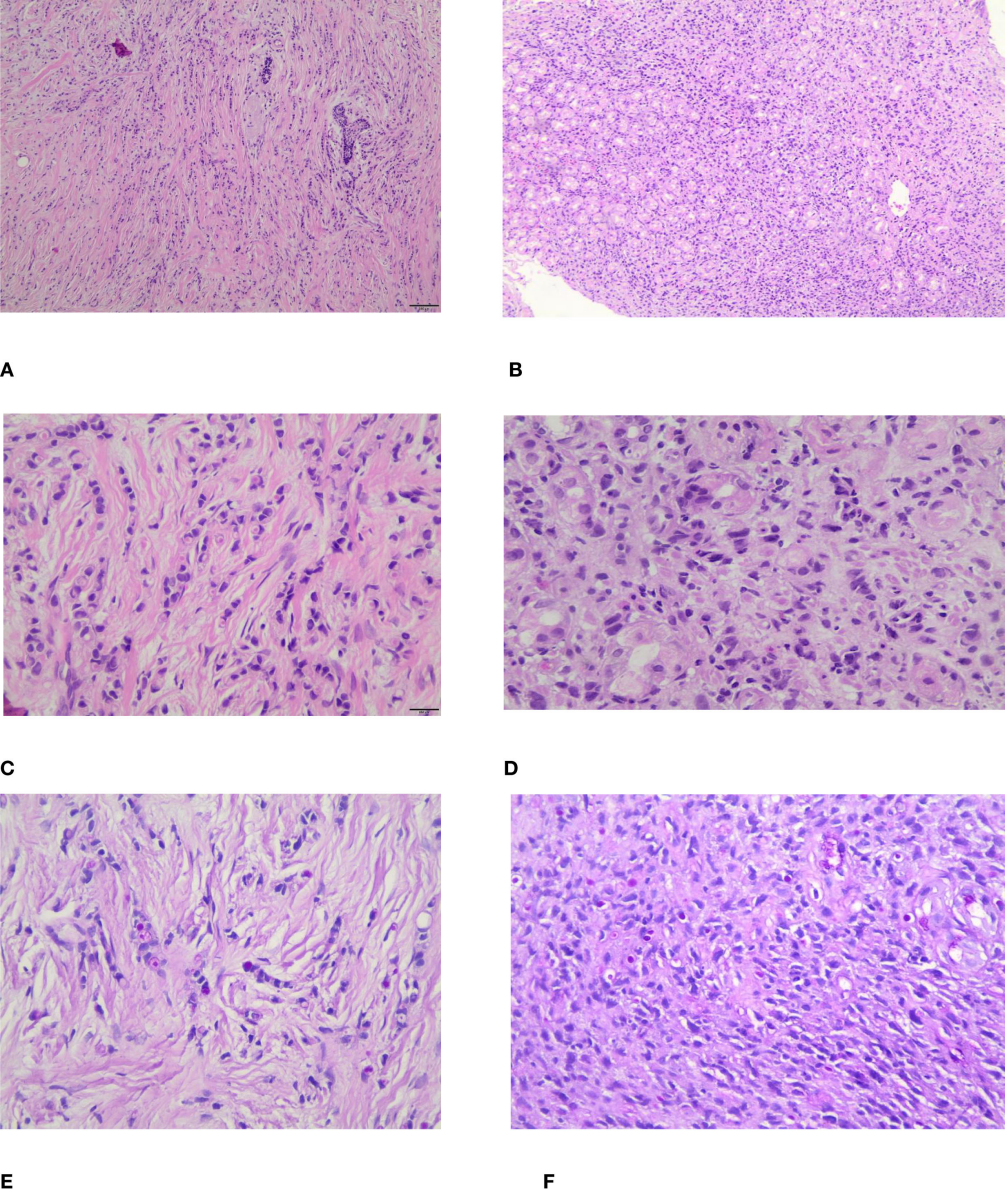
**(A-F)** Pathology images. At medium magnification, distortion of the normal breast **(A)** and stomach **(B)** tissue architecture by neoplastic cells with a linear and diffuse growth pattern is evident (*H&E, x100 magnification*). At higher magnification, neoplastic cells infiltrating the breast **(C)** and stomach **(D)** exhibit mild to moderate nuclear atypia, with several signet ring cells present (*H&E, x400 magnification*). The presence of intracellular mucin within the neoplastic cells in the breast **(E)** and stomach **(F)** was confirmed by PAS-D staining (*H&E, x400 magnification*).

**Figure 3:**
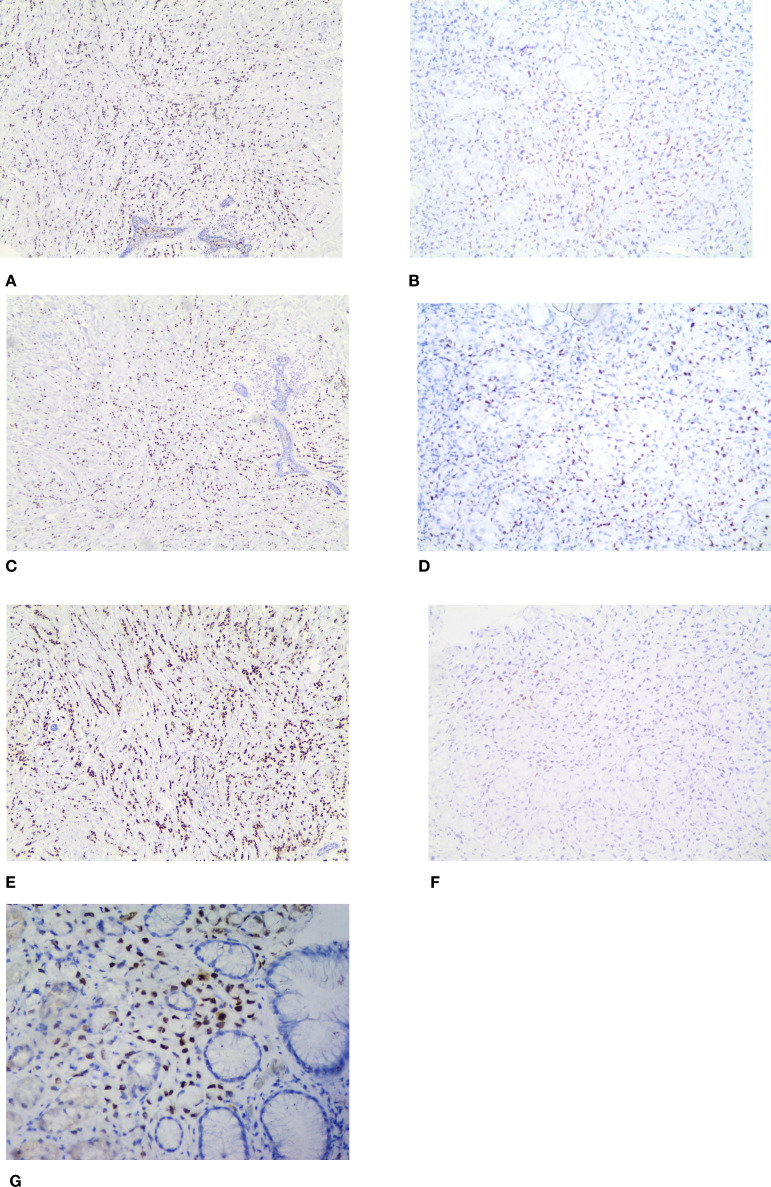
**(A-G)** GATA-3 positivity is shown in breast **(A)** and stomach **(B)** neoplastic tissue. ER positivity is shown in the breast **(C)** and stomach **(D)**. PR expression is demonstrated in the breast **(E)** and stomach **(F)**. [**(A, C, E)**: *x100 magnification*, **(B, D, F)**: *x200 magnification*]. **(G)** The neoplastic cells in the stomach were positive for TRPS1 (*x200 magnification*). Antigen retrieval was performed by heating slides in pH 9, in a PT instrument. The antibody used was the Rabbit Monoclonal TRPS1 (clone EPR16171, Diagnostic Biosystems), at a dilution of 1:60.

In December 2025, the patient reported epigastric discomfort and pain of two months’ duration and rising tumour markers (Ca 15-3 = 61.2U/mL, Ca 125 = 79.2U/mL). The patient underwent gastroduodenal endoscopy, which revealed markedly erythematous gastric mucosa with focal whitish areas, as shown in **Figure 1**. Gastric biopsies were interpreted as diffuse-type adenocarcinoma per Lauren classification with a subset of signet ring cells. IHC markers were positive for cytokeratin 8/18 and negative for E-cadherin and ER, raising suspicion for primary gastric carcinoma. Given the oncologic history and the potential therapeutic implications, a second external expert review was requested. Comparative review with the primary breast tumour and extended IHC with additional staining demonstrated tumour cell positivity for GATA3, TRPS1, ER (90%), and PR (95%), with HER2 score 0 and Ki-67 10%; E-cadherin and CDX2 were negative (**Figures 2**, **3**). This profile favored gastric metastasis from the patient’s known ILC. A chest–abdomen CT performed after endoscopy did not demonstrate clear radiologic progression. Nevertheless, due to persistent symptoms and weight loss, second-line systemic therapy with weekly paclitaxel was initiated.

**Table 1** provides an overview of the case presentation summary.

**Table 1:** Case presentation summary.

Parameter	Initial diagnosis (2022)	Progression of disease (2025)
Presenting Symptoms	Malignant ascites	Epigastric discomfort
Biopsy Location	Breast tissue	Gastric mucosa
Histopathological Findings	Invasive Lobular Carcinoma	Signet-ring adenocarcinoma
Immunohistochemistry	GATA-3+, ER+, PR+, HER2 0, PAX-8−, p53−, WT-1−, CDX-2−, CEA−, calretinin−, Ki-67 5%	GATA3+, TRPS1+, ER+, PR+, HER2 0, E-cadherin−, CDX2−, Ki-67 10%
CT Findings	Bone metastases, Peritoneal Implantations	No findings of progressive disease
Treatment	1^st^ line: carboplatin/paclitaxel, Maintenance: Ribociclib/GnRH analog	2^nd^ line: Paclitaxel

## 3 Discussion

The incidence rate of GI metastases originating from breast cancer is low, accounting for about 0.34% of cases (7). GI metastases from breast cancer are rare but clinically important, particularly in ILC, which exhibits a higher likelihood of GI dissemination than invasive ductal carcinoma (3) and it may emerge several years after the initial diagnosis (8). Presenting symptoms are obscure and they frequently overlap with GI diseases, such as gastritis (9). Nausea, vomiting, epigastric pain and dyspepsia are non-specific GI symptoms that have been observed, while intestinal obstruction has also been described in the literature (10).

Accurate diagnosis of GI metastases based solely on endoscopic appearance remains challenging in patients with ILC of the breast (11). Consequently, histopathological evaluation plays a pivotal role in establishing the correct diagnosis. Two main metastatic patterns have been described in the literature: linitis plastica–like infiltration and signet ring cell morphology. Linitis plastica has an infiltrative growth pattern frequently appearing as uniform, symmetrical and circumferential thickened gastric mucosal parts with a constricted lumen, which is far distinct from mass-like lesions. Rigidity and decreased peristaltic activity are also observed. On imaging it often presents as smooth bowel wall thickening (12). In published series, linitis plastica–like involvement has been reported in 73%–83% of cases of gastric metastases from breast cancer (13, 14). Notably, in a case series including 31 patients with linitis plastica secondary to metastatic breast cancer, all cases were ultimately attributed to ILC, highlighting the strong association between this histologic subtype and diffuse gastric infiltration (15).

On the other hand, signet ring carcinoma is a poorly differentiated aggressive subtype of adenocarcinoma mainly deriving from the stomach, but every organ could be a potential source of origin (16). Indeed, signet ring morphology represents a rare entity of ILC metastasis to the gastric mucosa. Reports on this entity are scarce and often restricted to single case reports published in the literature (17, 18). The rarity of this presentation underscores the importance of early recognition and careful pathological evaluation.

Typically, ILC with signet ring morphology exhibits a luminal A molecular phenotype and predominantly expresses ER and PR, while lacking HER2 expression. Notably, this subtype often displays a relatively low Ki-67 index. Despite these characteristics, ILC with signet ring morphology demonstrates distinct biological behavior compared to ILC without signet ring morphology. Studies have shown that patients with ILC containing 10% or more signet ring cells are at a higher risk of disease recurrence or metastasis compared to those with fewer than 10% (6). However, in our case presentation the patient did not exhibit signet ring morphology at the time of diagnosis. Consequently, in clinical settings, it is vital to quantify signet ring cells in ILC rather than overlook their presence.

Distinguishing metastatic breast carcinoma from primary gastric carcinoma is of critical clinical importance, as the therapeutic approach differs substantially. Primary gastric cancer often requires surgical resection when feasible, whereas metastatic breast cancer requires systemic treatment. Misinterpretation of metastatic ILC as a primary gastric malignancy may lead to unnecessary surgical intervention, potentially delaying the initiation of systemic treatment and allowing further disease progression. Such diagnostic pitfalls have been previously documented in the literature (18).

Morphological pathology evaluation using hematoxylin & eosin (H&E) stains and thus, analyzing extracellular mucin, architectural motives, and the cytoplasmic mucin/vacuole ratio, represents an important step to determine the cancer site of origin in signet ring adenocarcinomas (19). However, gastric and breast carcinomas can exhibit overlapping histomorphological characteristics, which may complicate this distinction (19). IHC is pivotal in differentiating primary gastric cancer from breast cancer metastasis. ILC is usually ER and PR positive and HER2 negative (20). Detection of ER and PR expression in a gastric biopsy specimen should therefore raise suspicion for metastatic breast carcinoma (21, 22). However, it should be highlighted that the loss of ER/PR expression in metastatic lesions does not exclude a carcinoma of breast origin. ER and PR status may change several times during metastatic tumor progression. Phenotypic drift is a common occurrence where tumor cells become more aggressive, dedifferentiate and lose receptor expression during disease progression (23–25). Evidently, hormone receptor positivity is not a reliable marker at differentiating primary gastric carcinoma from ILC metastatic disease.

Additional immunohistochemical markers have therefore been proposed to improve diagnostic accuracy (26). Loss of E-cadherin expression is indicative of ILC (27). GATA-3 is a transcription factor involved in key cellular processes, including differentiation, proliferation, and apoptosis (28). Its dysregulation is implicated in the epithelial differentiation of breast and urothelial carcinomas and its expression is useful in the diagnosis of breast and urothelial neoplasms, whether primary or metastatic (29). Another marker, gross cystic disease fluid protein-15 (GCDFP-15), is a glycoprotein associated with apocrine differentiation and may support a breast origin, although its sensitivity is lower than that of GATA-3 (30). Belonging to the GATA transcription factor family, TRPS1 is a zinc-finger transcription factor involved in breast epithelial cell regulation (31). It is a highly sensitive and specific marker for breast carcinoma and can be used as a great diagnostic tool (32). On the other hand, the nuclear transcription factor CDX-2, a pivotal factor in intestinal embryonic development, is a marker frequently detected in the signet ring carcinoma of the stomach (33). Additionally, GI signet ring cell carcinomas are usually positive for the epithelial marker cytokeratin (CK) 20, which is less observed with ILC specimens (34). Consequently, a recommended IHC panel for suspected gastric metastasis from breast carcinoma versus primary gastric carcinoma should include ER, PR, HER2, E-cadherin, GATA-3, GCDFP-15, TRPS1, CDX-2, CK-20 staining.

When the GI tract is involved secondary to ILC, the stomach and the small bowel are the most common sites affected, followed by the colon and the rectum (15). Metastatic involvement to the colon has also been reported, including a case of mixed IDC and ILC presenting with a linitis plastica–like morphology (35). In addition, another case of ILC metastasizing to the colonic mucosa has been described (36). Colonic involvement appears to be considerably rarer than gastric metastasis. Importantly, metastatic dissemination from ILC may affect any segment of the gastrointestinal tract, extending from the tongue to the anus (16).

Even more uncommon presentations have been reported. For instance, ILC metastasis to the pancreas mimicking primary pancreatic signet ring cell carcinoma has been documented (37). Similarly, a recent case described ILC metastasis to the urinary bladder presenting with signet ring cell morphology (38).

These rare and unusual cases emphasize an important diagnostic principle: not all tumours displaying signet ring morphology originate from the stomach.

## 4 Conclusion

Metastatic ILC may frequently mimic primary gastric signet ring cell carcinoma, rendering early and accurate diagnosis particularly challenging. Despite substantial overlap in clinical presentation, endoscopic appearance, and histopathological features, distinguishing between primary gastric carcinoma and metastatic breast cancer is of critical importance, as the therapeutic strategies and prognostic implications differ significantly.

Although this metastatic pattern of ILC has been previously described in the literature, the currently available evidence is largely limited to isolated case reports and small case series, which may contribute to its underrecognition in routine clinical practice by both pathologists and medical oncologists. In this context, IHC plays a pivotal role in resolving this diagnostic dilemma, providing essential information that facilitates accurate tumour origin identification, guides appropriate therapeutic decision-making, and ultimately contributes to improved patient management and outcomes.

## Data Availability

The original contributions presented in the study are included in the article/supplementary material. Further inquiries can be directed to the corresponding author.
